# TET1 is an important transcriptional activator of TNFα expression in macrophages

**DOI:** 10.1371/journal.pone.0218551

**Published:** 2019-06-19

**Authors:** Fangfang Sun, Irene Abreu-Rodriguez, Shuang Ye, Steffen Gay, Oliver Distler, Michel Neidhart, Emmanuel Karouzakis

**Affiliations:** 1 Department of Rheumatology, Center of Experimental Rheumatology, University Hospital Zurich, University of Zurich, Zurich, Switzerland; 2 Department of Rheumatology, South Campus, Ren Ji Hospital, School of Medicine, Shanghai Jiao Tong University, Shanghai, China; Auburn University - Harrison School of Pharmacy, UNITED STATES

## Abstract

Activation of macrophages and overexpression of TNFα is associated with the pathogenesis of chronic inflammatory diseases. However, the mechanisms leading to TNFα overexpression are still unknown. 5-methylocytosine (5-mC) is an epigenetic modification that is associated with silenced genes. Recent studies showed that it is converted to 5-hydroxylmethylocytosine (5-hmC) and reactivates gene expression through the action of the family of Ten-Eleven-Translocation (TET1-3) enzymes. In this study, we show that 5-hmC levels are increased globally and specifically in the TNFα promoter during the differentiation of monocytes to macrophages. In addition, the levels of 5-hmC are increased upon LPS stimulation of macrophages. Furthermore, CRIPSR stable knockout of TET1 decreases the expression of TNFα and other pro-inflammatory cytokines. In conclusion, we showed that TET1 contributes to the activation of macrophages possibly through regulation of 5-hydroxymethylation in the promoter of pro-inflammatory cytokine genes. The TET1 enzyme could be a promising therapeutic target to inhibit the persistent inflammation caused by macrophages in chronic inflammatory diseases.

## Introduction

Macrophages are members of the phagocytosis system[1]. Other members include monocytes, dendritic cells (DC), neutrophils, B cells and mast cells. They express TLRs receptors and variety of receptors facilitating phagocytosis. Macrophages are classified into two classes M1 and M2 depending on their function. M1 macrophages are considered important for defense against pathogens and anti-tumor immunity. In contrast, M2 macrophages have an anti-inflammatory role and express high levels of interleukin-10 (IL-10). The M1 macrophages are implicated in autoimmune diseases by different studies[2,3]. For example, rheumatoid arthritis (RA) synovial macrophages are the main TNFα producing cells in the inflamed synovium[4]. This cytokine has been therapeutically targeted with successful results in patients with RA. However, the mechanism of periodical overproduction of TNFα, associated with disease flares, is still unknown.

Few studies have studied the role of epigenetic control at the TNFα locus in cell lines and primary cells. DNA methylation has shown to regulate TNFα gene transcription. Thus, analysis of the TNFαpromoter in three human cell lines showed that there is a high level of DNA demethylation in the cells expressing high levels of TNFα[5]. In addition, demethylation of the TNFα promoter in cell lines using the DNMT1 inhibitor 5-azacytidine increased the transcription of TNFα[5].

Recent findings have shown that 5-methlcytosine (5-mC) modification of DNA can be converted to 5-hydroxymethylcytosine (5-hmC) through the activation of the family of Ten-Eleven-Translocation (TET1-3) enzymes, which play an important role in the active DNA demethylation pathway[6]. TET can further oxidize 5-hmC to 5-formylcytosine and 5-carboxylcytosine. These two oxidized forms, recognized by thymine DNA glycosylase, can be converted back to 5-cytosine through the so-called active demethylation pathways[7]. Alternatively, 5-mC can be diluted through DNA replication by a passive demethylation process, as this occurs in RA synovial fibroblasts[8].

TET enzymes were firstly discovered to be involved in the differentiation of embryonic stem cells[9]. Recent evidence showed that the TET2 enzyme regulates the differentiation of Tregs [10] and the cytokine expression of T cells[11]. In particular, TET2 has shown to reduce inflammation by repressing the expression of IL-6 [12] and to play a role in the differentiation of human monocytes to dendritic cells. In addition, TET2 overexpression can cause myeloid malignancies, while reduced expression of TET genes as well as 5-hmC was also observed in cancers, especially colon cancer[13].

Here we aim to investigate the 5-hmC modification and DNA demethylation pathway mediated by TET enzymes in macrophages stimulated with lipopolysaccharide (LPS). It is shown for the first time that 5-hmC and the enzyme TET1 contribute to the activation of macrophages through the regulation of 5-hydroxymethylation at the TNFα promoter.

## Materials and methods

### Cell culture

The human monocytic leukemia cell line THP-1 (Cell Lines Service GmbH) was cultured in RPMI supplemented with 10% fetal calf serum (FCS, Life Technologies, Basel, Switzerland) and used below passage 10. THP-1 cells were differentiated into macrophages in the presence of 50 ng/ml phorbol myristate acetate (PMA, Sigma, USA) for 48 hours. THP-1 PMA-derived macrophages were stimulated with 10 ng/ml E. coli LPS (List Biological Laboratories, California) for 2 hours. In other experiments, PBMCs were isolated from buffy coats. The buffy coats were obtained from the Zurich Red Cross blood center in which written informed consents and procedures were approved by the Kanton Zurich ethical commission under the BASEC-Nr 2019–00115 application agreement. Monocytes were isolated by CD14 positive magnetic beads isolation and differentiated with 10 ng/ml macrophage colony stimulating factor (M-CSF) in RPMI with 10% fetal calf serum (FCS) for 6 days.

### siRNA knockout

THP-1 PMA-differentiated macrophages were transfected with scrambled and TET 1,2,3 siRNAs (Qiagen, Netherlands) using TransIT-X2 dynamic delivery system (Mirus Bio, USA), then stimulated with 10 ng/ml LPS.

### Lentivirus CRISPR plasmid construction

Three different guide RNAs (sgRNAs) for TET1 knockdown (Table 1) were designed using the online program ChopChop (http://chopchop.cbu.uib.no/) and cloned to a Mule Entry Vector (plasmid 62127, Addgene USA). Cloning of sgRNAs to the vectors was modified from Albers et. al.[14]. The forward and reverse oligonucleotides that encode the sgRNAs include the restriction-cutting site of Bfua1 and not the PAM sequences. They were annealed by reducing the temperature from 95 to 10°C by programming the thermocycler to ramp down 3°C per 30 sec. In the meantime, the Mule entry vector have been digested with BfuA1. The predigested vector was ligated with the sgRNA oligos using a mixture of T4 ligase buffer and T4 ligase for 30 min at room temperature. Next, the cloned plasmids were transformed into competent E. coli according to manufacture protocol. Positive clones that contain the sgRNA sequences were cultured overnight and a Midiprep was used to purify the sgRNA plasmid DNA. Last, each sgRNA vectors and a Mule CAS9 vector (plasmid 62134, Addgene) were recombined into a Lentiviral GFP destination vector (plasmid 62175, Addgene). A 2-fragment recombination reaction was performed using the Gateway LR Clonase II Plus Enzyme Mix (12538, Invitrogen USA) according to manufacture protocol. Then, two μl of the above reaction was used to transform On Shot Mach1 T1 competent E. coli and the bacteria were incubated on ampicillin resistant plates.

**Table 1:** sgRNA sequences used for CRISPR targeting of TET1.

sgRNA target	Target sequence 5' -sgRNA-PAM-3'
Scrambled control	GTCATGTCACTTATCAAGTC
TET1 Exon 4	ACAAAGTTCATGCAACACGG **TGG**

### Lentivirus production and THP1 cell transduction

Lentiviruses were produced in HEK293T cells transfected using PEI (1 μg/μl) with the lentiviral CRISPR TET1 vector together with the viral packaging psPAX2 and envelope p MD2G constucts. Viral supernatants were collected after 24 and 48 hours post-transfection. The supernatant were filtered and used for THP1 cell transduction unconcentrated. The lentiviral particles were diluted two times with RPMI and mixed with 8 μg/μl polybrene. After 72 hours incubation with the virus, the transduced cells were washed three times and analysed for GFP expression by flow cytometry. Single GFP positive cells were sorted into a 96 well plate using a BD FACS Aria II Cell sorter. Single cell clones were expanded in cell culture and assayed with mismatch cleavage assay.

### T7 Endonuclease I mismatch cleavage assay

T7 Endonuclease I assay was used to detect CRISPR mutations in the TET1 locus. Thereafter, the cells were expanded from a cell clone. Genomic DNA was extracted using the Qiagen blood kit. PCR was set up and run using specific PCR primers that amplify the TET1 exon 2 sgRNA target site (Table 2). Next, the PCR product (200 ng or 10 μl) was denatured at 95°C and annealed by slowly reducing the temperature by 2°C per second from 95 to 25°C using a thermocycler. The annealed PCR product was incubated with 5 units/μl of T7 endonuclease I at 37°C for 15 minutes. Last, the reaction was stopped by adding 1 μl of 0.5M EDTA and fragment analysis was performed by agarose electrophoresis.

**Table 2:** Sequences of primers used for TI7 Endonuclease assay.

Primer	Sequence (5'-3')	Product Size (bp)
TET1 Exon 4 forward TET1 Exon 4 reverse	TGCAAACCATAAAAACGCTATG TATTTCTTGGTGGCAACTGATG	737

### RT-qPCR

Total RNA was extracted with Relia PrepTM RNA Cell Miniprep System (Promega, USA) and reverse transcribed to cDNA with reverse transcriptase (Applied Biosystems, USA). Quantitative PCR reaction was performed with SYBR green (Roche, Switzerland) on Taqman 7500 Real-time PCR system (Applied Biosystems). Exon spanning specific primers were designed using Primer-BLAST (Table 1). Cycle thresholds (Ct) were normalised to the housekeeping gene RLPO.

The differences of the comparative threshold cycles (Ct) of sample and RLPO cDNA were calculated (dCt). Relative expression levels were calculated following the formula ddCt = dCt (sample stimulated)—dCt (sample unstimulated). Finally, the amount of target gene was calculated using the mathematical formula 2^-ddCt^.

### DNA dot blot

DNA dot blots were performed to determine the global 5-hmC levels. Genomic DNA was purified using QIAmp DNA kit (Qiagen). 200ng genomic DNA were diluted with 0.1M NaOH and denatured at 95°C for 5 minutes. After cooling down on ice and neutralised with 6.6 M ammonium acetate, DNA samples were spotted on the Amersham Hybond-N+ nylon transfer membrane (GE Healthcare Life Science, USA). After UV-x-link, the membrane was blocked with fat-free 10% milk overnight at 4°C and then incubated with 5-hmC antibodies for 1 hour. Global 5-hmC was detected with ECL Plus Western Blotting Reagent Pack (GE Healthcare) and quantified using the BioRad image analysis software.

### Hydroxymethylated DNA immunoprecipitation

Hydroxymethylated DNA immunoprecipitation (hMeDIP) was performed to analyse the 5-hmC enrichment in the gene promoters. Genomic DNA was prepared by QIAamp DNA blood mini kit (Qiagen) and fragmented by sonication to a size range of 200-1000bp confirmed by gel electrophoresis. As a positive control for 5-hmC, we used together with our tested DNA 1μl of hydroxymethylated DNA of APC (adenomatous polyposis coli) promoter from the methylated DNA standard kit (Active Motif, USA). Denatured at 99°C for 10 minutes, 10% of the genomic DNA was collected as input, the rest was incubated with 1μl 5-hmC antibodies (Active Motif) at 4°C overnight. Dynabeads Protein A (Invitrogen / Thermo-Fisher, Switzerland) was applied to capture the hydroxymethylated DNA. Immunoprecipitated (IP) samples were released from the beads by resuspension in digestion buffer with protease K and incubation at 50°C for 2 hours in a shaking heat block at 800rpm. IP and input samples were purified by QIAquick PCR purification kit (Qiagen) and analysed by quantitative Real-time PCR in the Taqman 7900 PCR system (Applied Biosystems) using specific primers for the TNFαpromoter (Table 3). The enrichment was presented as ratio of IP copy number to input copy number.

**Table 3:** Primer sequences used for hydroxylmethylated DNA immunoprecipitation (hMeDIP).

Genes	F/R	Sequence
GAPDH	Forward	5’-CAA TTC CCC ATC TCA GTC GT-3’
	Reverse	5’- ACG CTT GGA TGA AAC AGG AG-3’
TNFA1	Forward	5’-CTC TCG CCC CAG GGA CAT AT-3’
	Reverse	5’- ATG TGG CGT CTG AGG GTT GT-3’
TNFA2	Forward	5’- CGC TTC CTC CAG ATG AGC TC-3’
	Reverse	5’- TGC TGT CCT TGC TGA GGG A-3’
TNFA3	Forward	5’- CCC CCT CGG AAT CGG A-3’
	Reverse	5’- GAG CTC ATC TGG AGG AAG CG-3’
TNFA4	Forward	5’- CCC AAA AGA AAT GGA GGC AAT-3’
	Reverse	5’- AAG CAT CAA GGA TAC CCC TCA C-3’

### Western blot

Nuclear and cytoplasmic extracts were prepared by NE-PER nuclear and cytoplasmic extraction reagents (Thermo Scientific) and quantified by Pierce BCA Protein Assay Kit (Life Technologies, USA). Samples, separated by 6% SDS-polycrylamide gel, were transferred to nitrocellulose membranes. The membranes were blocked with 3% bovine serum albumin at room temperature for 1 hour and incubated with TET1 antibodies (Active Motif) at 4°C overnight. TET1 proteins were visualized using ECL chemiluminescence (Amersham) and quantified by Image J software (NIH).

### ELISA

THP1 PMA derived macrophages were transfected with TET1-3 siRNA and simulated with LPS for 24 hours. Cell culture supernatants were measured in duplicates for TNFαand IL-8, using the Human TNFα and IL-8 ELISA set (BD Biosciences) according to the manufacturer’s instructions.

### Statistics

All data are expressed as mean ±SEM. Statistical analysis was performed using GraphPad Prism software, version 8.0 (GraphPad System, San Diego, CA). For analysis between the different experiments, the student t-test was used. *P* values less than 0.05 were considered significant.

## Results

### TET1 is active during macrophage differentiation and inflammation

**Fig 1:**
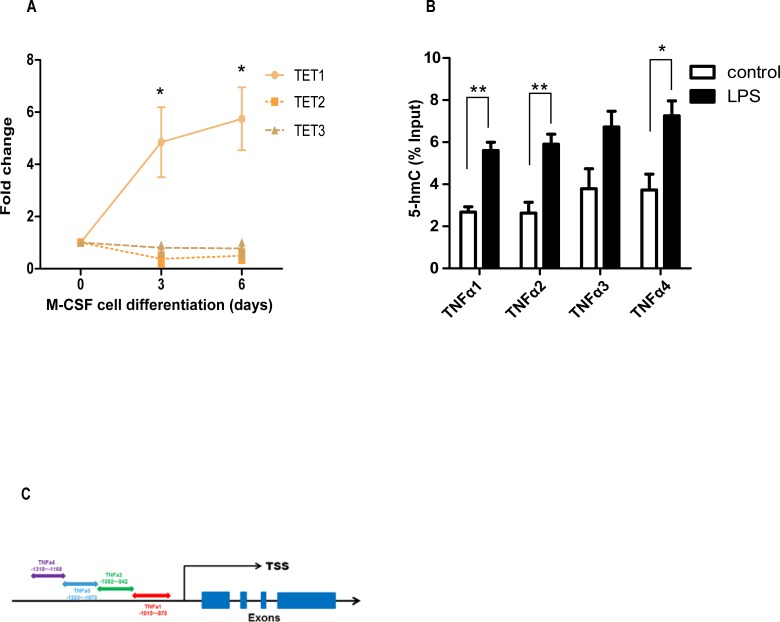
DNA demethylation pathway activated during macrophage differentiation and inflammation. (A) TET1 mRNA expression is significantly increased during the differentiation of monocyte-derived macrophages. Student’s t-test d3: TET1 * p = 0.01, d6: TET1 * p = 0.01. (B) hMeDIP showed that LPS stimulated monocyte-derived macrophages have more 5-hmC enrichment in the TNFαpromoter than unstimulated control macrophages. Data are representative of three independent experiments. Student’s t-test, TNFA1 ** p = 0.003, TNFA2 ** p = 0.009, TNFA3 p = 0.07, TNFA4 * p = 0.03, mean values and SE ± were calculated for each transcript and promoter region. (C) Four different promoter sites were analyzed upstream the TNFα transcription start site (TSS).

Since TET enzymes are involved in lymphoid and myeloid cell reprograming, we measured TET transcripts by real time PCR after M-CSF-induced differentiation of monocytes into macrophages. Interestingly, we observed an exponential increase in expression of TET1 mRNA (d3: 4.85, d6: 5.74 fold change) but not of TET2 (d3: 0.37, d6: 0.80 fold change) or TET3 (d3: 0.80, d6: 0.77 fold change) transcripts (Fig 1A, n = 3 per transcript). Since, LPS stimulated macrophages are potent producers of TNFα, we were interested to test the levels of 5-mC oxidation in the TNFα promoter under inflammatory conditions. We found a significant enrichment of 5-hmC modification in four different regions of TNFα promoter (Fig 1B and 1C TNFA1: control 2.7±0.25 LPS 5.7±0.39, TNFA2: control 2.6±0.51 LPS 5.9±0.48, TNFA3: control 3.8±0.94 LPS 6.8±0.75, TNFA4: control 3.7±0.75 LPS 7.2±0.70% input, n = 3 per promoter region). Overall, these data showed the importance of active DNA demethylation in TNFα regulation during inflammation.

### DNA demethylation is also active in THP1 PMA-differentiated macrophages

**Fig 2:**
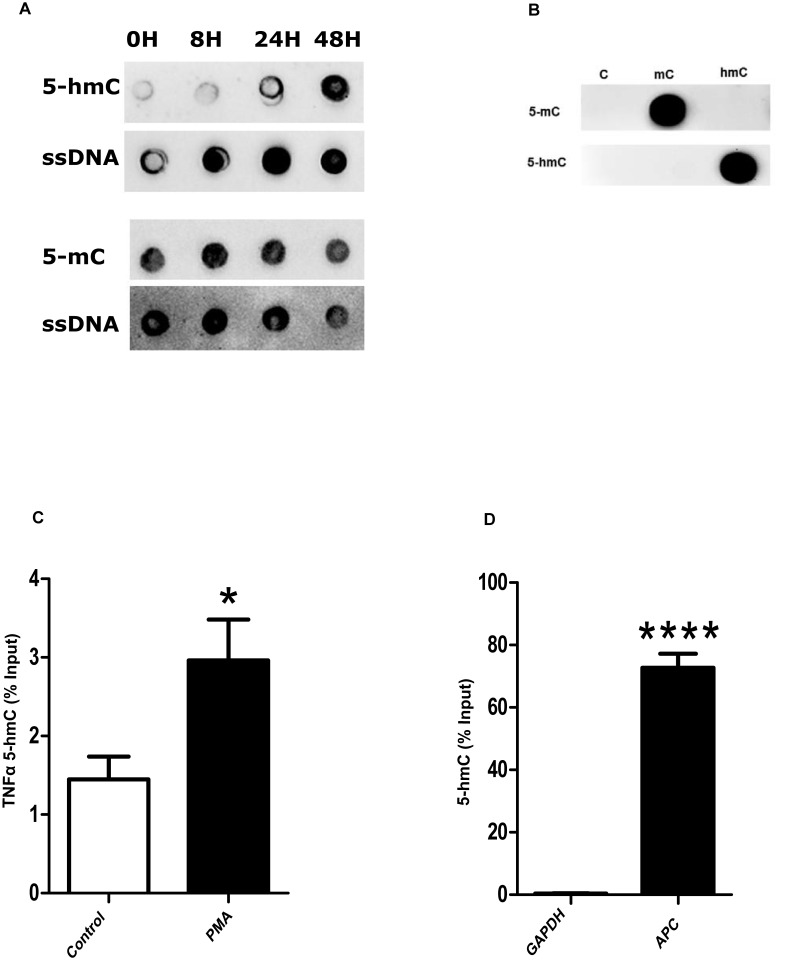
5-hmC is increased during THP1 macrophage differentiation. (A) THP1 monocytes were differentiated with PMA (50ng/ml) into macrophages after 24 hours. Dot blot assays were performed with 5-hmC antibodies using DNA at different time points of macrophage differentiation. 5-hmC modification was increased time-dependently. (B) The 5-hmC antibodies are specific for 5-hmC and did not detect 5-mC modifications. (C) Fully differentiated THP1 PMA macrophages have an increase in 5-hmC enrichment in the TNFα promoter. Student’s t-test, TNFA1 PMA * p = 0.03. (D) The in vitro hydroxylmethylated APC spike in DNA was used as positive, while GAPDH served as negative control. Data are representative of three independent experiments. Student’s t-test, APC **** p = 0.0001.

Regarding the importance of DNA demethylation in cell differentiation, we determined the global levels of 5-hmC in THP1 cells upon PMA differentiation at different time points (0h, 8h, 24h and 48h). The undifferentiated monocytes (THP-1) had the lowest levels of 5-hmC. The highest levels of 5-hmC occurred upon 24 hours of PMA stimulation when the monocytes have differentiated into macrophages (Fig 2A and 2B). Furthermore, we searched for changes in 5-hmC in the promoter of TNFαand found by hMeDIP a significant increase in differentiated macrophages (Fig 2C TNFA1: control 1.4±0.29 PMA 2.9±0.51% input, n = 6, Fig 2D GAPDH 0.39±0.06 APC 72±4.5% input, n = 3).

### LPS induces 5-hmC in TNFα promoter of THP1 PMA-differentiated macrophages

**Fig 3:**
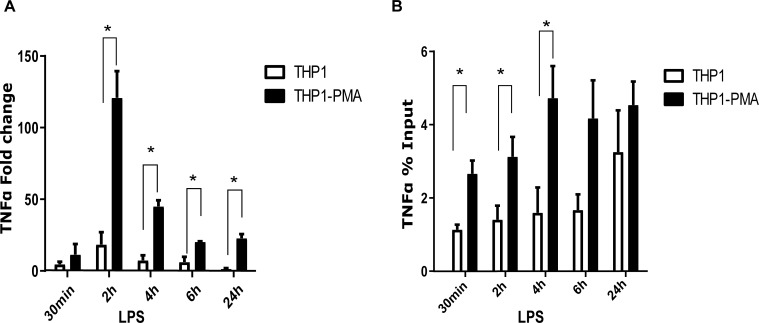
5-hmC is increased during THP1 macrophage stimulation. LPS stimulation (10 ng/ml) of THP1 PMA-differentiated macrophages induces a time dependent increase of 5-hmC modification in the TNFα promoter. (A) Stimulated THP1 macrophages expressed higher mRNA levels of TNFα than the undifferentiated monocytes, shown as fold change increase of TNFα mRNA. Student’s t-test, TNFA1 30min: p = 0.46, 2h: * p = 0.03, 4h: * p = 0.02, 6h: * p = 0.05, 24h * p = 0.01. (B) 5-hmC is significantly more enriched in TNFα promoter of stimulated THP1 PMA macrophages than the undifferentiated monocytes. Data are representative of three independent experiments. Student’s t-test, TNFA1 30min: * p = 0.01, 2h: * p = 0.05, 4h: * p = 0.04, 6h: p = 0.09, 24h p = 0.38.

Next, we compared mRNA expression levels of pro-inflammatory cytokines between monocyte and macrophages using LPS as inflammatory stimulus. THP1 PMA-differentiated macrophages were as expected stronger responders of TNFα than the monocytes (Fig 3A TNFα 30min: THP1 4.6±1.7 PMA 11.5±7.4, 2h: THP1 18.4±8.6 PMA 120±18.5, 4h: THP1 7.4±3.5 PMA 45±4.2, 6h: THP1 6±3.6 PMA 20±0.4, 24h: THP1 1.5±0.4 PMA 23±2.8 fold change, n = 2 per time point). During each stimulation time point, we measured the 5-hmC modification by hMeDIP and found a time dependent increase in the level of 5-hmC especially for the TNFα promoter. Interestingly, the 5-hmC enrichment was significantly higher in macrophages than in monocytes (Fig 3B TNFA1 30min: THP1 1.13±0.13 PMA 2.66±0.36, 2h: THP1 1.4±0.38 PMA 3.2±0.53, 4h: THP1 1.6±0.69 PMA 4.8±0.87, 6h: THP1 1.67±0.43 PMA 4.17±1.0, 24h: THP1 3.25±1.13 PMA 4.5±0.64% Input, n = 3 per time point).

### TET1 regulates the mRNA and protein expression of TNFα in THP1 PMA-differentiated macrophages

**Fig 4:**
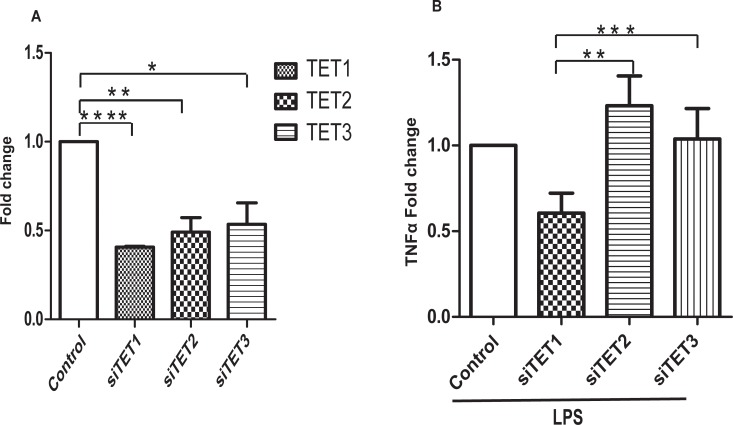
TET1 regulates the mRNA and protein expression of TNFα. **(**A) We measured the fold change of gene expression of TET genes for each siRNA tested. Specific siRNAs for TET enzymes efficiently downregulated the mRNA of TET1, 2, 3 respectively in THP1 PMA macrophages. Student’s t-test **** p<0.0001, ** p = 0.03, * p = 0.01 (B) THP1 PMA macrophages were transfected with control and TET1, 2, 3 siRNAs before being stimulated with LPS (10 ng/ml) for 2 hours. TET1 but not TET2, 3 siRNAs reduces the mRNA fold change of TNFα in THP1 derived macrophages. Data are representative of three independent experiments. Student’s t-test ** p = 0.002, *** p = 0.0009.

**Fig 5:**
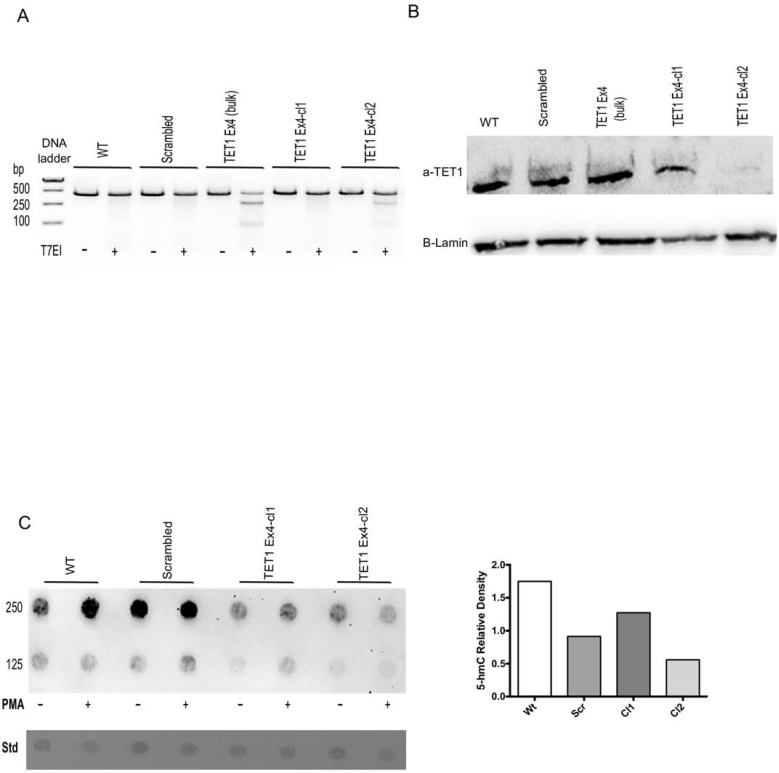
THP1 TET1 knockout cell line was generated using CRISPR/Cas9. **(**A) THP1 cells were transduced with lentiviruses expressing Cas9 and sgRNA against TET1 ex4 and a non-specific sgRNA (scrambled-control). Untransfected (WT) and scrambled cells were used as controls. After GFP cell sorting and culturing, genomic DNA was isolated from bulk and single cell clones. PCR amplification of the region targeting by CRISPR were subjected to T7 Endonuclease I (T7EI). The appearance of cleaved products in sgRNA transduced cells but not in WT or scrambled cells indicate mutation of the targeted locus. (B) Nuclear extracts from the indicative CRISPR targeting THP1 cells were analyzed using an antibody for TET1 by Western blot. Absence of TET1 protein was observed in the monoclonal line (cl2). (C) Equal amounts of genomic DNA were spotted to a nitrocellulose membrane (Std) and 5-hmC relative density using a 5hmC antibody using the dot blot method as previously (right). Densitometry analysis of the upper lane dots (250ng) revealed reduced global levels of 5-hmC in the sgRNA TET1 ex4 cl2 in comparison to the controls (left).

**Fig 6:**
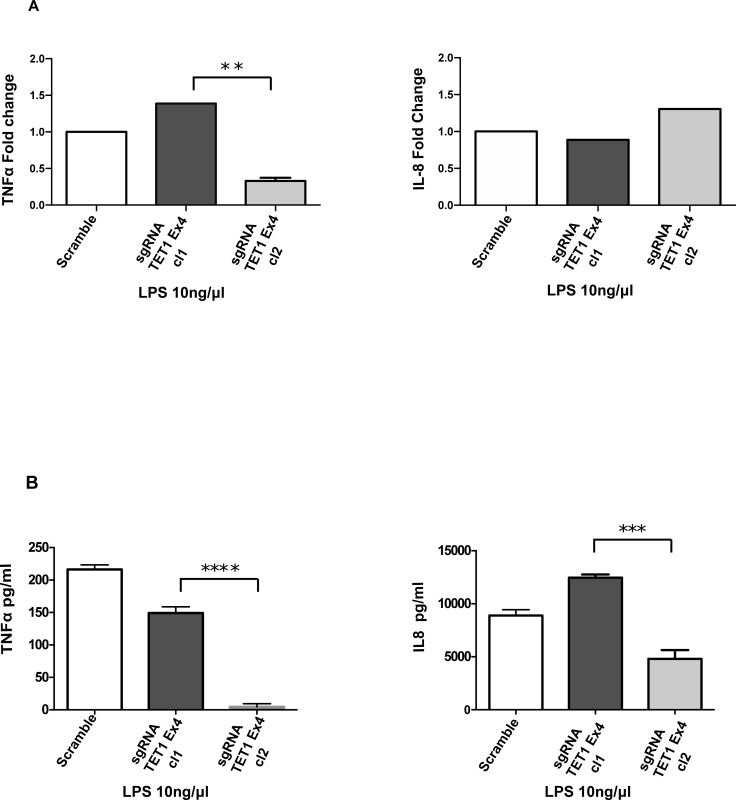
Effect of LPS in CRISPR TET1 knockout PMA derived macrophages. (A) THP1 PMA-differentiated macrophages expressing Cas9 with sgRNA for TET1 ex4 cl-1, scramble and sgRNA for TET1 ex4 cl2 were stimulated with LPS. The expression of TNFα and IL-8 was analyzed by Real-time PCR. The sgRNA TET1 ex4 cl2 knockout monoclonal cell line downregulate significantly TNFα gene expression but not IL8. Student’s t-test, TNFα **p = 0.003. (B) Supernatants were collected from the same experimental conditions. Upon LPS, levels of TNFα and IL-8 in the cell culture supernatant were significantly reduced in the knockout PMA-differentiated macrophages (sgRNA TET1 ex4 cl2). Data are representative of three independent experiments. Student’s t-test, TNFα**** p<0.0001, IL-8 ***p = 0.0001.

In order to better understand the role of 5-hmC in the regulation of TNFα expression, we knocked-out the expression of TET enzymes with siRNA in THP1 PMA-differentiated macrophages (Fig 4A TET1: control 1, siTET1 0.40±0.005 TET2: control 1, siTET2 0.49±0.08 TET3: control 1, siTET3 0.53±0.12 fold change, n = 3). siTET1, 2, 3 knockdown THP1 PMA-differentiated macrophages were stimulated with LPS. Interestingly, only TET1 inhibition reduced the stimulatory capacity of macrophages as shown by the significantly reduced mRNA and protein levels of TNFα (Fig 4B TNFα: 0.40±0.005 fold change, n = 3). In contract, siTET2 and siTET3 did not reduce the expression of TNFα transcripts (Fig 4B TNFα: 1.10 ±0.09 and 1.07±0.07 fold change, n = 3). To further analyze the role of TET1, we created a stable THP1 TET1 knockout cell line using the CRISPR methodology. Three different sgRNAs were designed targeting three different TET1 exons sequences. The sgRNA TET1 exon 4 was able to induce a mutation in TET1 genomic DNA as shown by T7 endonuclease I assay (Fig 5A). A Western blot of nuclear lysates extracted from the knockout cell line showed the absence of TET1 protein (Fig 5B). The global levels of 5hmC were reduced between control and knockout THP1 PMA-differentiated macrophages, as shown by dot blot analysis (Fig 5C). Last, the TET1 knockdown macrophages were stimulated with LPS and we found that the mRNA and protein expression of TNFα was significantly reduced in the knockout macrophages (Fig 6A TNFα: scramble 1, sgRNA TET1 ex4 cl1 1.38±0.1, sgRNA TET1 ex4 cl2 0.32±0.02 fold change. Fig 6B TNFα: scramble 216±4, sgRNA TET1 ex4 cl1, 149±5 sgRNA TET1 ex4 cl2 4.5±2.7 pg/ml, n = 3). In contrast, other pro-inflammatory cytokines such as IL-8 were not affected at the transcriptional, but only at the protein level (Fig 6A IL8: scramble 1, sgRNA TET1 ex4 cl1: 0.89±0.1, sgRNA TET1 ex4 cl2: 1.3±0.04 fold change. Fig 6B IL8: scramble 8.9±0.3, sgRNA TET1 ex4 cl1 12.5±0.2, sgRNA TET1 ex4 cl2 4.8±0.5 μg/ml, n = 3).

## Discussion

DNA methylation modifications play an important role in the differentiation and plasticity program of myeloid and lymphoid cells[15–17]. In this study, we further extended our analysis on the epigenetic control of macrophages TNFα promoter under differentiation and inflammatory conditions.

Previous studies have shown that TET2 has an important role in the myeloid lineage. We measured the expression of TET genes and observed an upregulation of TET1 transcripts during macrophage differentiation induced by M-CSF. Since the enzyme regulates the oxidation of 5-mC to 5-hmC, we searched for potential genes with high 5-hmC enrichment during stimulation with an inflammatory stimulus such as LPS. Interestingly, we identified TNFα as such a gene, which is a relevant therapeutic target in RA, ankylosing spondylitis and inflammatory bowel diseases. Then, we further analyzed the role of the DNA demethylation pathway in the regulation of TNFα transcription in the human monocyte THP1 cell line.

THP1 cells are known to differentiate into macrophages *in vitro* using PMA as a stimulant, and express high levels of TNFα. Therefore, we searched for global changes of the 5-hmC and 5-mC modifications using THP1 monocytes differentiated with PMA for 48 hours. Interestingly, we observed an increase of 5-hmC during the macrophage differentiation process. We could reverse the upregulation of 5-hmC in the CRISPR TET1 knockout cell line and this further support the role of this enzyme in macrophage differentiation. Similarly, this increase of 5-hmC has also been detected in mouse embryonic stem cell and immune cell differentiation processes[10, 18–20]. Since changes of 5-hmC affect gene promoters and enhancers, we searched for the occurrence of 5-hmC in the promoter of TNFα which has been previously described to have low levels of methylation [5] and strongly expressed in THP1 cells. Our results showed a strong enrichment in 5-hmC levels of TNFα cytokine during differentiation. This effect is not unexpected because other reports have shown that T cell cytokines such as IL-4, INFγ and IL-17 have enriched 5-hmC depending on the T cell subset that expresses these cytokines[11,21]. The demethylation of CpG sites in the promoter or regulatory regions has also been described in Treg cell differentiation. Three intrinsic enhancers of Foxp3 transcription factor are enriched with 5-hmC during Treg cell differentiation[10].

Macrophages are key cells in the pathogenesis of chronic inflammatory diseases. During disease progression, these cells can react to various stimuli such as PAMPs, DAMPs, immune complexes and other immune cells. These pathways stimulate macrophages to produce pro-inflammatory cytokines such as TNFα. In the current study, we mimicked the inflammatory stimuli using LPS and hypothesized that the demethylation of the TNFα promoter occurred during inflammation. Interestingly, we observed that macrophages have higher enrichment of 5-hmC during LPS stimulation and conclude that this epigenetic modification increases TNFα expression. It is known from genome wide 5-hmC map experiments and transcriptomic studies that 5-hmC enrichment correlates with gene expression[22].

In order of find the causative factor for the increase in 5-hmC, we silenced the TET enzymes separately and measured TNFα expression. In addition, we created a stable TET1 knockout cell line that confirmed with reductions of TNFα mRNA and protein expression. These experiments revealed that TET1, but not TET2 or TET3, is a regulator of TNFα expression and possibly of other pro-inflammatory cytokines. Furthermore, these results confirmed the observation of previous reports that showed no change in TNFα gene expression in the TET2 knockdown murine bone marrow macrophages[12]. Therefore, our results propose that TET enzymes have distinct functions in the regulation of pro-inflammatory cytokines.

Interestingly, the CRISPR TET1 stable knockout line IL8 transcription was not affected but only the release into cell culture supernatant. The TET1 knockout has shown in mouse embryonic stem cells (mESCs) to affect the upregulation and downregulation of multiple genes[23]. Therefore, there is a possibility that TET1 affects the IL-8 release indirectly through the upregulation of various mechanisms. A RNA sequencing experiment using the TET1 knockout cell line can identify potential novel factors, which regulate the expression and/or release of other proinflammatory cytokines.

In conclusion, TET1 enzyme is an important target of inflammatory macrophages and could provide a new therapeutic target for diseases such as RA and other inflammatory diseases.
